# Binge-Pattern Alcohol Exposure during Puberty Induces Long-Term Changes in HPA Axis Reactivity

**DOI:** 10.1371/journal.pone.0018350

**Published:** 2011-04-13

**Authors:** Magdalena M. Przybycien-Szymanska, Natasha N. Mott, Caitlin R. Paul, Roberta A. Gillespie, Toni R. Pak

**Affiliations:** Department of Cell and Molecular Physiology, Stritch School of Medicine, Loyola University Chicago, Chicago, Illinois, United States of America; Medical College of Georgia, United States of America

## Abstract

Adolescence is a dynamic and important period of brain development however, little is known about the long-term neurobiological consequences of alcohol consumption during puberty. Our previous studies showed that binge-pattern ethanol (EtOH) treatment during pubertal development negatively dysregulated the responsiveness of the hypothalamo-pituitary-adrenal (HPA) axis, as manifested by alterations in corticotrophin-releasing hormone (CRH), arginine vasopressin (AVP), and corticosterone (CORT) during this time period. Thus, the primary goal of this study was to determine whether these observed changes in important central regulators of the stress response were permanent or transient. In this study, juvenile male Wistar rats were treated with a binge-pattern EtOH treatment paradigm or saline alone for 8 days. The animals were left undisturbed until adulthood when they received a second round of treatments consisting of saline alone, a single dose of EtOH, or a second binge-pattern treatment paradigm. The results showed that pubertal binge-pattern EtOH exposure induced striking long-lasting alterations of many HPA axis parameters. Overall, our data provide strong evidence that binge-pattern EtOH exposure during pubertal maturation has long-term detrimental effects for the healthy development of the HPA axis.

## Introduction

Alcohol abuse, especially binge-pattern alcohol abuse, is highly prevalent among adolescents. According to the Substance Abuse and Mental Health Services Administration, 36% of youths ages 18 to 20 reported at least one binge-drinking episode during the past 30 days, defined by National Institute on Alcohol Abuse and Alcoholism (NIAAA) as consuming four or five drinks within a two hour period that causes a rapid increase in blood alcohol concentration (BAC) to the level of 0.08 g % or greater. Our previous studies demonstrated that binge-pattern EtOH exposure in adolescent rats negatively affected the HPA axis by dysregulating normal negative feedback pathways, however whether these neurobiological changes were transient or persistent remained unclear [1]. Thus, a central focus of this study was to determine the long-term neurobiological consequences of adolescent binge-pattern alcohol exposure on adult HPA axis reactivity. The HPA axis is extremely plastic during pubertal development, suggesting that early alcohol abuse could permanently disrupt the normal maturational process of the HPA axis.

Alcohol is a physiological stressor and potent activator of the HPA axis [1], [2], [3], [4]. Acute psychological or physical stressors activate parvocellular neurons in the hypothalamic paraventricular nucleus (pPVN) to release corticotrophin releasing hormone (CRH) and arginine vasopressin (AVP) into the portal system of the anterior pituitary gland, which, upon stimulation, releases adrenocorticotrophic hormone (ACTH). ACTH then acts on the adrenal cortex to release glucocorticoids, cortisol in humans and corticosterone (CORT) in rodents, into the systemic circulation setting up a negative feedback loop by which CORT acts on the hypothalamus and pituitary gland to decrease further release of CRH, AVP, and ACTH. During pubertal development, the brain and HPA axis undergo extensive maturational processing, characterized by altered HPA axis reactivity, increased synaptic connections, and differential responsiveness to the effects of gonadal steroid hormones [5], [6], [7], [8], [9]. These studies indicate that the successful shaping of mature HPA function that occurs during pubertal development may be extremely vulnerable to the detrimental effects of alcohol.

We have previously shown that binge-pattern EtOH exposure in peri-pubertal male rats increased the expression of CRH and AVP mRNA in the PVN during this time period [1], however the long lasting effects of this pattern of EtOH exposure during puberty on the PVN and the HPA axis as a whole remain unknown. In this study we tested the hypothesis that binge-pattern EtOH exposure during puberty has long lasting effects on the reactivity of the adult HPA axis. The overall goals of this study were to 1) determine how binge-pattern EtOH exposure during puberty affects central expression of adult CRH and AVP, as well as circulating CORT levels, and 2) determine whether adults pre-exposed to EtOH during puberty were more sensitive to subsequent EtOH exposures in adulthood. Overall, our results showed that peri-pubertal binge-pattern EtOH exposure induced a striking long lasting alteration of many HPA axis parameters. Our data provide strong evidence that binge-pattern EtOH exposure during pubertal maturation has long-term detrimental effects for the healthy development of the HPA axis.

## Materials and Methods

### Ethics Statement

All animal procedures were approved by the Loyola University Medical Center Institutional Animal Care and Use Committee (IACUC) permit #2007027.

### Animals

Male Wistar rats were purchased from Charles River Laboratories (Wilmington, MA) at weaning (postnatal day (PND) 23) and allowed to acclimate for 7 days after arrival. Animals were handled for 5 min once/day beginning at PND 30. Pubertal EtOH treatments began on PND 37 which is defined as peri-puberty [10], [11], [12]. Animals were undisturbed following pubertal EtOH treatments until adult EtOH treatments began at PND 68 (early adulthood). Animals were pair-housed on a 12:12 light/dark cycle with lights on at 07.00 h. Food and water were available *ad libitum.*

### Binge Exposure Paradigm and Treatment Design

After one week of daily handling, animals at PND 37 were randomly assigned to either 1) saline-treated (N = 30) or 2) binge EtOH treated (N = 30, 3g/kg/day) groups. The animals in the binge EtOH group received an intraperitoneal (IP) 20% (v/v in saline) EtOH injection every morning at 10:00 AM for 3 consecutive days, followed by 2 days of saline injections, and then an additional 3 days with EtOH. This binge exposure paradigm has been used previously to mimic the pattern of binge alcohol consumption in adolescents [1], [13] and it has been shown that in pubertal animals this IP route of alcohol administration does not yield different blood alcohol concentrations (BAC) compared to oral gavage nor does it disrupt normal growth rates [1], [13], [14]. The saline group was treated with IP saline (0.9%) injection each day for 8 consecutive days. After pubertal treatments, animals were undisturbed until PND 68 when each group was divided into following treatment groups (see Table 1): 1) saline only control group (N = 10); 2) acute EtOH group (N = 10, one IP saline injection each day for 7 consecutive days, followed by one IP EtOH (3 g/kg) injection on day 8) and 3) binge EtOH group (N = 10). On the last day of treatment (PND 75), animals were euthanized by rapid decapitation 60 min after the final injection.

**Table 1:** Experimental Design.

Puberty (PND 37–44)	Adult (PND 68–75)
	Saline (N = 10)
Saline (N = 30) “EtOH Naïve”	Acute EtOH (N = 10)
	Binge EtOH (N = 10)
	Saline (N = 10)
Binge EtOH (N = 30) “EtOH pre-exposed”	Acute EtOH (N = 10)
	Binge EtOH (N = 10)

Diagram describing the animal treatment groups. Left column indicates treatment the animals received during puberty (saline  =  EtOH naïve, or Binge EtOH  =  EtOH pre-exposed). Left column indicates the subsequent treatments the same animals received as adult (saline, acute EtOH, Binge EtOH). PND  =  post natal day.

### Blood alcohol concentration (BAC) measurements

Trunk blood samples were collected into heparinized tubes, centrifuged at 3000 rpm for 10 min at 4°C; and plasma stored at −20°C. Blood alcohol levels were determined by measuring the change in absorbance at 340 nm following enzymatic oxidation of EtOH to acetylaldehyde (Point Scientific Alcohol Reagent Kit). Assay range is 0 to 400 mg/dl and intra and interassay CV = 6.4% and 7.9%, respectively.

### CORT measurements

Plasma corticosterone (CORT) was measured using radioimmunoassay (RIA) as previously described [1]. Briefly, blood samples were collected into heparinized tubes, centrifuged at 3000 rpm for 10 min at 4°C and plasma stored at −20°C. To measure CORT, ^3^H-CORT (PerkinElmer, Waltham, MA) and rabbit CORT antiserum (MP Biomedicals, Solon, OH) were used to perform RIA. On the first day of the procedure, a standard curve was prepared using CORT-standards (4-PREGNEN, 11b, 21-DIOL-3,20-DIONE-Steraloids, Inc., Newport, RI). Appropriate sample tubes were filled with 100 µl of a sample, 100 µl of antibody solution (in 0.1% gel PBS), and 100 µl diluted radioactive tracer (10,000–12,000 cpm). Tubes were incubated overnight at 4°C. On the second day, all tubes were filled with 100 µl of 0.5% gel PBS, 1 ml Dextran-Coated Charcoal (DCC), and 1.0 ml 0.01 M PBS. Samples were centrifuged at 3000 rpm at 4°C for 15 min and the supernatant was collected into scintillation vials. Radioactivity was measured in each vial for 3 min using a Packard liquid scintillation counter. Levels of CORT in unknown samples were interpolated based on the standard curve. Intra and interassay CV were 7.9% and 14.6%, respectively.

### Tissue collection and qRT-PCR

After sacrifice, brains were rapidly collected and frozen using isopentane and stored at −80°C until further processing. Tissue collection and qRT-PCR were performed as previously reported [1]. Briefly, frozen brains were sectioned at 200 µm on a freezing microtome and the paraventricular (PVN) and supraoptic (SON) nuclei were microdissected using a 0.75 mm Palkovit's brainpunch tool (Stoelting Co., Wood Dale, IL). The specificity of the microdissected regions was confirmed using The Rat Brain in Stereotaxic Coordinates, Fourth Edition Atlas (G. Paxinos and C. Watson). For the PVN, we microdissected 0.75 mm area on each side of the third ventricle between 0.8 mm and 2.12 mm posterior to Bregma 8 mm below the top of the brain [15]. For the SON we microdissected a 0.4 mm area 9.5 mm below the top of the brain between 0.8 mm and 3.14 mm posterior to Bregma. Total RNA isolation was performed on sonicated tissue samples using Trizol reagent (Invitrogen Inc., Carlsbad, CA) according to the manufacturer's directions. Following RNA isolation, 0.5 µg total RNA was reverse transcribed using the First Strand Synthesis SuperMix for qRT-PCR (Invitrogen Inc., Carlsbad, CA). Roche FastStart SYBR Green Master Mix was added to intron-spanning AVP specific upper and lower AVP primer (0.25 µM final concentration; 5-GGGCAGGTAGTTCTCCTCCT; 5-CACCTCTGCCTGCTACTTCC) and intron-spanning CRH primer (0.25 µM final concentration; 5-GAGAAAGGGGAAAGGCAAAG; 5-ATCAGAATCGGCTGAGGTTG). Then, 2 µL cDNA templates were added to duplicate reactions performed in 96 well plates. Quantification of the target gene expression was achieved by extrapolating from a standard curve of known concentrations of AVP or CRH ran simultaneously in the same plate. All samples were normalized to the hypoxanthine guanine phosphoribosyl transferase 1 (HPRT) housekeeping gene, as it is not altered by EtOH treatment [1].

### Statistical Analysis

Statistical analyses were performed by the Biostatistics Core Facility at Loyola University Stritch School of Medicine in consultation with Dr. James Sinacore. Two-way ANOVA was used to test for interactions between pubertal and adult treatments and for main effects of these treatments in regards to the following dependent variables: plasma BAC, plasma CORT levels, CRH mRNA in the PVN, and AVP mRNA in both the PVN and SON. Tukey's post hoc test was used if ANOVA achieved significance. If ANOVA showed interactions between treatments, Student's t-tests were used to assess significance between alcohol and saline pre-exposed groups within specific adult alcohol treatments. All tests were performed using SigmaStat Statistical Analysis Software. A p-value of less than 0.05 was considered to be significant.

## Results

### BAC following adult acute and binge EtOH exposure

BACs were measured on the final day of adult treatment 60 min following the injections. There were no statistically significant differences in BAC between either acute (205.1±21.8; 221.8±19.1 mg/dl for EtOH naïve and EtOH pre-exposed groups, respectively) or binge EtOH treated groups (172.9±27.0; 208.2±28.2 mg/dl for EtOH naïve and EtOH pre-exposed groups, respectively). BAC in animals treated with saline were below the limit of detection for the assay. Interestingly, prior EtOH exposure (i.e. EtOH pre-exposed binge group) had no effect on adult BAC after either acute or binge EtOH treatments. The values observed in EtOH-treated groups are consistent with the defined BAC threshold for binge drinking (80 mg/dl) and showed that all EtOH-treated groups were intoxicated to the same degree.

### Binge-pattern EtOH exposure during puberty significantly altered adult CORT levels

**Figure 1:**
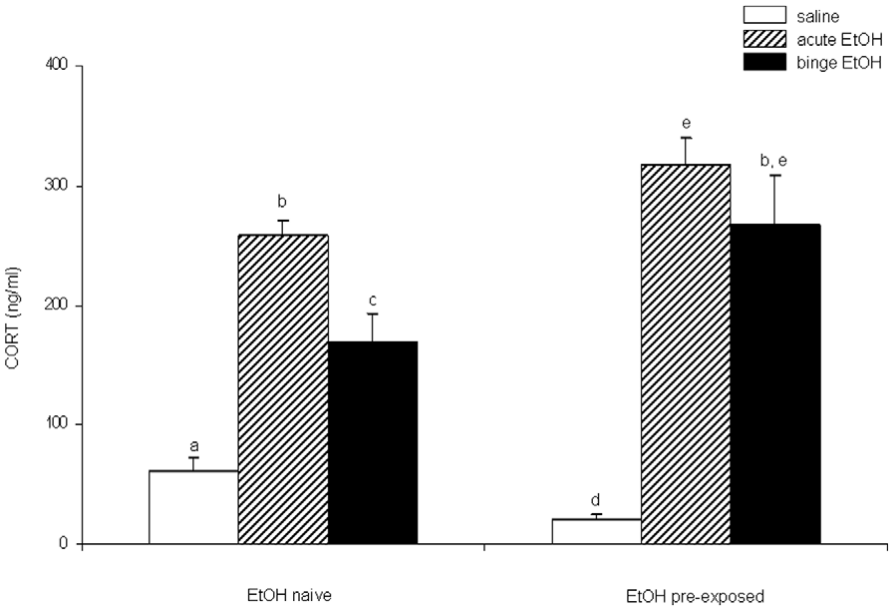
Effects of pubertal binge ethanol pre-treatments on plasma CORT levels in EtOH exposed adult animals. Plasma corticosterone (CORT) levels 1.0 h post-injection of 3 g/kg EtOH in adult animals. Animals were treated during puberty with either saline (EtOH Naïve) or binge EtOH (EtOH pre-exposed). Adult animals were then treated with saline (open bars), acute EtOH (hatched bars) or binge EtOH (solid bars). Data are expressed as mean ± SEM CORT ng/ml. Dissimilar letters indicate a statistically significant difference between groups for all pairwise comparisons (P<0.05).

Circulating CORT levels were measured by RIA to determine whether binge EtOH exposure during puberty affected responsiveness of the HPA axis to subsequent EtOH exposures in adulthood. All measurements were taken in adult animals and they were classified as either EtOH pre-exposed or EtOH naïve. Three different measures of adult HPA reactivity, as ascertained from plasma CORT levels, were compared: 1) basal pre-stress, 2) sensitivity to a single EtOH exposure (acute stress), and 3) sensitivity to repeated EtOH exposure (binge – repeated stress). Two-factor ANOVA was used to assess main effects of pubertal and adult treatments and interactions between these two factors. Every parameter measured was significantly different between animals that were EtOH pre-exposed compared with those that only received saline during puberty (EtOH naïve). There was a statistically significant main effect of EtOH treatment during adulthood (F(2,51)  = 71.218, p<0.001), no significant main effect of treatment during puberty (F(1,51)  = 3.305, p = 0.075) and a there was a significant interaction between pubertal and adult treatment (F(2, 51)  = 4.458, p = 0.016). Overall, our data revealed that the pattern of plasma CORT responses were significantly different depending on whether the animals were pre-exposed to EtOH (Fig. 1). Notably, basal levels of plasma CORT were significantly lower in animals that had previously been exposed to EtOH during pubertal development compared with EtOH naïve animals (t(17)  = 3.23, p = 0.005, Fig. 1, open bars). Second, plasma CORT levels in animals that were pre-exposed to EtOH during puberty were significantly higher following adult treatment with a single (acute) (t(18)  =  −2.247, p = 0.037) or binge-pattern EtOH (t(15)  =  −2.218, p = 0.042) (Fig. 1, hatched and solid bars), suggesting that the adult animals were more sensitive, or that the HPA axis was potentially more responsive, to the stressful effects of EtOH. Animals that did not have prior EtOH exposure also had significantly elevated CORT levels following EtOH treatment in adulthood (F(2, 27)  = 34.105, p<0.001), but as mentioned above, it was significantly lower than the animals that were pre-exposed. Finally, animals that were pre-exposed to EtOH during puberty failed to habituate to the binge-treatment paradigm as indicated by their equivalently higher plasma CORT levels following a subsequent acute or binge EtOH treatment (p = 0.358, Fig. 1). By stark contrast, animals that received saline during puberty (EtOH naïve) did show a habituation effect to the repeated binge EtOH treatment (p = 0.003), which is consistent with our previous reports in peri-pubertal animals.

### Binge-pattern EtOH exposure during puberty significantly altered responsiveness of adult CRH and AVP mRNA in the PVN to subsequent EtOH exposure

**Figure 2:**
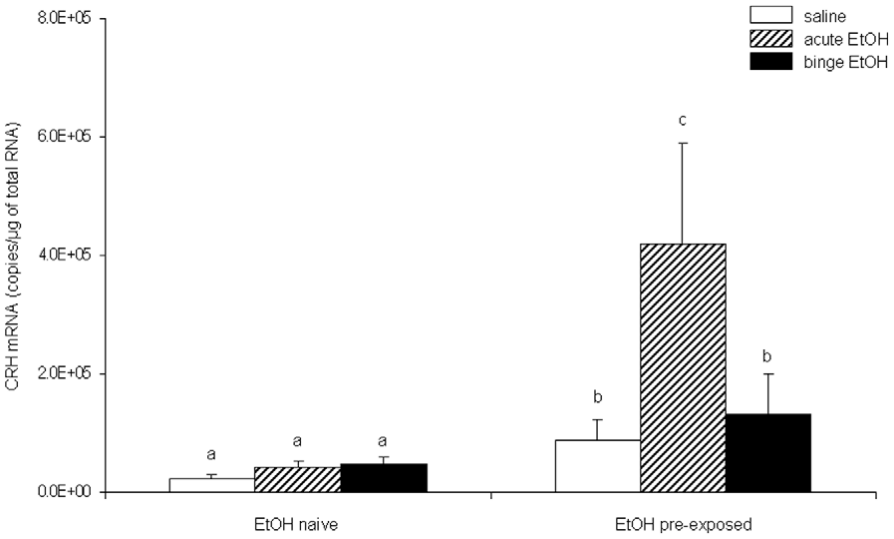
Effects of pubertal binge ethanol pre-treatments on CRH gene expression in the PVN of ethanol exposed adult rats. CRH mRNA expression 1.0 h post-injection of 3 g/kg EtOH in adult animals. Animals were treated during puberty with either saline (EtOH Naïve) or binge EtOH (EtOH pre-exposed). Adult animals were then treated with saline (open bars), acute EtOH (hatched bars) or binge EtOH (solid bars). Data are expressed as mean ± SEM CRH mRNA copies/µg total RNA. Dissimilar letters indicate a statistically significant difference between groups for all pairwise comparisons (P<0.05).

**Figure 3:**
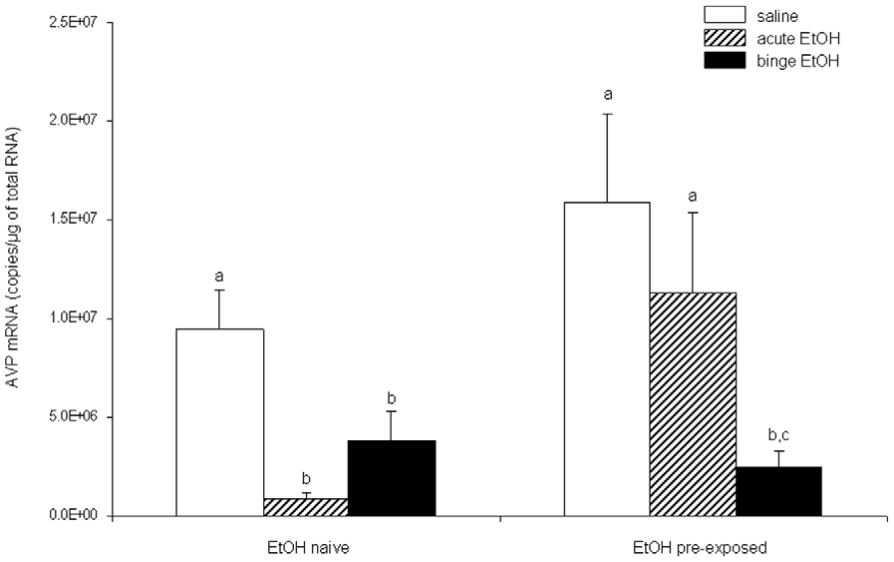
Effects of pubertal binge ethanol pre-treatments on AVP gene expression in the PVN of ethanol exposed adult rats. AVP mRNA expression 1.0 h post-injection of 3 g/kg EtOH in adult animals. Animals were treated during puberty with either saline (EtOH Naïve) or binge EtOH (EtOH pre-exposed). Adult animals were then treated with saline (open bars), acute EtOH (hatched bars) or binge EtOH (solid bars). Data are expressed as mean ± SEM AVP mRNA copies/µg total RNA. Dissimilar letters indicate a statistically significant difference between groups for all pairwise comparisons (P<0.05).

Next, we measured CRH and AVP mRNA in the PVN using quantitative RT-PCR to determine the long-term effects of pubertal binge EtOH exposure on adult HPA axis reactivity. CRH and AVP mRNA were used as endpoints to ascertain three different measures of adult HPA axis status: 1) basal pre-stress, 2) sensitivity to a single EtOH exposure (acute stress), and 3) sensitivity to repeated EtOH exposure (binge – repeated stress). Two-way AVOVA showed that there was a main effect of EtOH treatment during puberty on adult CRH mRNA expression (F(1, 42)  = 8.641, p = 0.005), as well as a main effect of pubertal treatment (F(1, 44)  = 4.876, p = 0.032) and adult treatment (F(2, 44)  = 5.835, p = 0.006) for AVP mRNA expression in the PVN (Figs. 2, 3). Similar to the results obtained with plasma CORT levels, the patterns of gene expression for CRH and AVP were significantly different depending on whether the animals were pre-exposed to EtOH or EtOH naïve.

Basal pre-stress levels of CRH mRNA were significantly higher in animals that were exposed to EtOH during puberty compared with those that received saline (EtOH naïve) (t(14)  = −2.175, p = 0.047) (Fig. 2, open bars). A single (acute) dose of EtOH significantly increased CRH mRNA levels (t(14)  =  −2.363, p = 0.033) in animals that were pre-exposed to EtOH during adolescence, yet had no effect in animals that were EtOH naïve (Fig. 2, hatched bars). Conversely, binge-pattern EtOH exposure significantly increased CRH mRNA levels in EtOH pre-exposed animals compared to EtOH naive, however the levels were not statistically different from the baseline levels of EtOH pre-exposed animals (Fig. 2, solid bars).

Pre-exposure to EtOH during puberty had no effect on baseline levels of adult AVP mRNA (t(15)  =  −1.342, p = 0.199, Fig. 3, open bars). However compared to baseline, a single (acute) dose significantly decreased AVP mRNA levels in EtOH naïve animals (t(15)  =  −2.214, p = 0.043), but had no significant effect compared to baseline in animals pre-exposed to EtOH (Fig. 3, hatched bars). Binge-pattern EtOH exposure significantly decreased AVP mRNA levels compared to baseline in both groups, however the levels were only different from acute EtOH treatment in the pre-exposed group (Fig. 3, solid bars).

### Binge EtOH exposure during puberty does not change the expression of the AVP mRNA in adult SON

**Figure 4:**
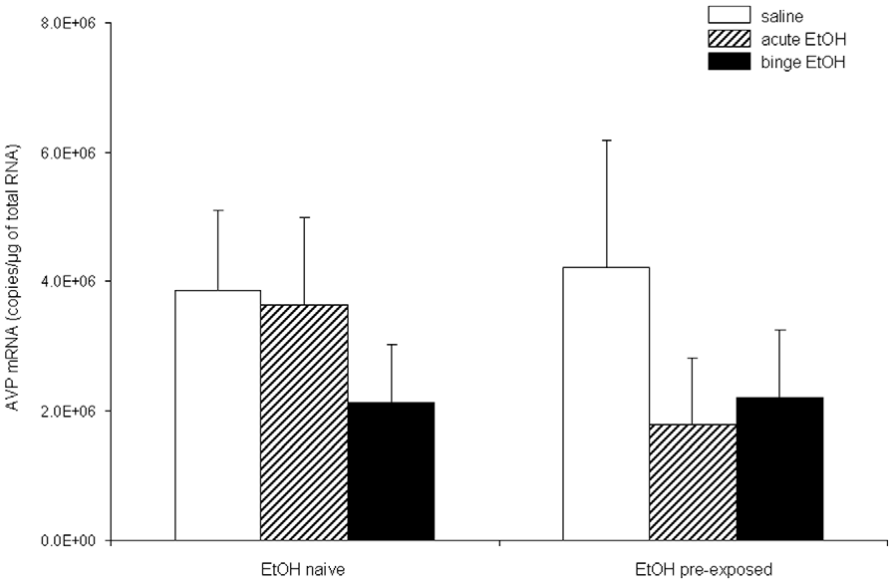
Effects of pubertal binge ethanol pre-treatments on AVP gene expression in the SON of ethanol exposed adult rats. AVP mRNA expression 1.0 h post-injection of 3 g/kg EtOH in adult animals. Animals were treated during puberty with either saline (EtOH Naïve) or binge EtOH (EtOH pre-exposed). Adult animals were then treated with saline (open bars), acute EtOH (hatched bars) or binge EtOH (solid bars). Data are expressed as mean ± SEM AVP mRNA copies/µg total RNA. Dissimilar letters indicate a statistically significant difference between groups for all pairwise comparisons (P<0.05).

To confirm that the observed changes in the expression of the AVP mRNA in PVN after EtOH treatments were not due to the reported diuretic effects of alcohol, we measured AVP mRNA expression in the supraoptic nucleus of the hypothalamus (SON), a region responsible for maintaining water homeostasis. Two way AVOVA showed no main effects of either pubertal (F(1, 48) = 0.65, p = 0.424) or adult (F(2, 48)  = 1.72, p = 0.19) treatment and no significant interactions between treatments during puberty and during adulthood (F(2,48)  = 1.359, p = 0.267) (Fig. 4).

## Discussion

The goals of this study were to 1) identify the long lasting effects of binge EtOH exposure during pubertal development on the maturation of the HPA axis by measuring circulating CORT, and hypothalamic neuropeptides (CRH and AVP), previously identified to be susceptible to EtOH effects and 2) determine whether binge-pattern EtOH exposure during puberty renders the adult HPA axis more sensitive, or responsive, to subsequent EtOH exposure. The most striking finding of our study was that exposure to binge-pattern EtOH during puberty resulted in a permanent dysregulation of the adult HPA axis, which is consistent with our hypothesis and is substantiated by the following data. First, adult animals that were previously exposed to binge-pattern EtOH during puberty had significantly lower circulating basal CORT levels, yet increased basal CRH mRNA expression in the PVN. We previously showed that peri-pubertal animals exposed to binge-pattern EtOH had significantly elevated levels of CRH and AVP mRNA in the PVN [1] and the data herein demonstrate that those observed elevated CRH levels are maintained well into adulthood. Second, binge EtOH exposure during puberty sensitized the adult HPA axis to subsequent stressful stimuli (acute EtOH exposure), evident by a marked increase in adult levels of CRH mRNA in the PVN and higher circulating CORT levels compared to controls following a single exposure to EtOH. Finally, the stress response to EtOH exposure was significantly exacerbated and there was no habituation effect following repeated doses of EtOH in adults that were previously exposed to binge-pattern EtOH during pubertal development, which is contrary to the effects observed in adult EtOH naïve animals. Collectively, the data reported herein underscore the heightened reactivity of the adult HPA axis in animals previously exposed to alcohol during adolescence and suggest that EtOH might interfere with the normal development of the HPA axis.

EtOH is a potent activator of the stress response as indicated by increased plasma CORT levels after both acute and binge treatments, however repeated doses over several days (i.e. binge-pattern) leads to an habituation effect [1]. This same pattern has been observed with other types of psychological and physiological stressor (acute vs. chronic stress) in adult animals. Adult animals exhibit a high increase in CORT levels after exposure to a single predator stress however, after chronic homotypic stress this response is diminished [16]. Herein, we have shown that animals exposed to acute or binge EtOH only during adulthood had increased plasma CORT levels and, as observed in pubertal animals, this effect was diminished following a binge-pattern of EtOH exposure indicating habituation of the HPA axis to the repeated homotypic stressor. By contrast, adult animals that received prior EtOH exposure during puberty not only had higher plasma CORT levels, but the habituation effect was not observed. These data indicate that after binge EtOH exposure during puberty, the HPA axis is not only more sensitive to subsequent EtOH exposures but also that its normal ability to habituate to repeated stressors is compromised.

Our data are consistent with other studies that show adult EtOH exposure alters the expression of CRH and AVP in the PVN [2], [3], [17], [18]. For instance, adult rats treated with a single 3 g/kg EtOH dose had increased CRH and AVP mRNA in the PVN, whereas chronic dietary EtOH treatment reduced the number of AVP immunoreactive neurons in that same brain region [17]. Notably, the decreased AVP immunoreactivity was maintained 4 months after EtOH withdrawal in animals that received chronic dietary EtOH treatment, highlighting the potential for EtOH to exert a permanent effect on specific regulatory parameters of the HPA axis. We have shown previously that binge-pattern, but not acute, EtOH exposure during puberty increased both CRH and AVP mRNA in the PVN [1]. Contrary to what has been observed in other studies in adult EtOH naïve animals, [2], [19], [20], we did not observe an increase in the CRH mRNA levels in adult animals treated with acute EtOH exposure. This discrepancy is possibly due to differences in mRNA detection methods, as well as timing. In our study, we measured CRH and AVP mRNA levels one hour following the last EtOH injection. To our knowledge there are no other reports that have measured CRH and AVP gene expression this early following EtOH exposure. In this study, we show that this increased gene expression persists into adulthood regardless of subsequent EtOH exposure, although the molecular mechanisms mediating these long-term changes remain to be elucidated.

Recent evidence suggests that acute alcohol exposure can interfere directly with CRH promoter activity to alter CRH gene transcription [4]. For example, Li et al. showed that alcohol decreased forskolin-induced increase in CRH promoter activity possibly by interfering at the cAMP response element (CRE) site in the NO108-15 cell line [4]. In addition, recent evidence also suggests that EtOH can alter the DNA status [21] or glucocorticoid response element (GRE):DNA binding in different brain regions, including cortex and hippocampus, due to chromatin remodelling [22], raising the possibility that epigenetic mechanisms might underlie the EtOH-induced long-term changes in CRH and AVP gene expression. Histone deacetylases (HDAC) have the ability to remove acetyl groups off the chromatin making DNA more dense and unavailable for transcriptional machinery [23]. Interestingly, Pandey et al. showed that HDAC activity was decreased following acute EtOH exposure (single 1 g/kg injection of EtOH), yet increased after chronic EtOH exposure (15 days of 9% EtOH liquid diet) in adult animals [21]. One possibility is that binge-pattern EtOH exposure during puberty induces remodelling of the chromatin surrounding the CRH and/or AVP genes thereby permanently altering the access of transcriptional regulators for these gene promoters.

To our knowledge this is the first study showing the effects of adolescent binge EtOH exposure on adult HPA axis reactivity, however other reports have demonstrated that pubertal EtOH exposure has long lasting effects on a variety of other CNS systems, including the mesolimbic dopaminergic and glutaminergic system [24], hippocampus, and cerebellum [25]. Such changes in the CNS are often manifested by altered adult behaviour patterns [24], [25], [26]. For example, Pascual et al. showed that 2 weeks of intermittent EtOH administration (single IP injection of 3 g/kg EtOH for two consecutive days, followed by 2 day rest, then followed again by 2 days of EtOH injections) caused an increase in voluntary EtOH consumption in adult animals [24]. In a different study, they showed that this intermittent pattern of EtOH administration induced long lasting deficits in learning ability, as discerned by a decreased number of correct choices in conditional discrimination learning task, and a reduced ability to adapt to a challenging environment in adulthood in a narrow beam task [25]. Our data showing parallel effects of peri-pubertal binge EtOH exposure to the effects of juvenile stressors on the adult HPA axis [27] or juvenile/early puberty EtOH exposure on EtOH abuse in adulthood [24] indicate that during the peri-pubertal period the HPA axis is still immature and extremely vulnerable to EtOH-induced insults [28], [29].

Physiological parameters of HPA axis, such as CORT and neuropeptide levels, are markedly different in response to both acute and chronic stressors in juvenile compared with adult animals, suggesting that an extensive maturational process occurs during the pubertal transition [5], [6], [7], [8], [30], [31], [32]. Most striking is the observation that corticosterone and ACTH levels take much longer to return to baseline in juvenile compared to adult animals when subjected to a variety of stressful stimuli [5], [33]. Further, juveniles have higher overall stress reactivity [5] and chronic stress during puberty has been shown to result in an exaggerated stress-induced CORT response in adulthood [30], [34]. Our results are consistent with these observations, as our animals pre-exposed to binge-pattern EtOH had a similarly exaggerated response to a subsequent EtOH stressor during adulthood. It is important to note that there are some inconsistencies between studies depending on the type of stressor and the specific paradigm (i.e. homotypic vs. heterotypic, chronic vs. acute). Consequently, the effects of EtOH on the HPA axis during adolescence may not necessarily be reflective of other types of stressors. Nevertheless, these data contribute to the growing body of evidence that perturbations to the HPA axis during this critical period of pubertal development can potentially have long lasting consequences for the adult stress response.

We are confident that the effects observed here on the expression of the AVP mRNA are specific to changes in the population of cells located in the pPVN, and not to the magnocellular region. First, our data are consistent with our previous report showing no changes in the AVP mRNA expression in SON, a region largely responsible for maintaining osmotic homeostasis [1]. Second, our data are also consistent with other reports showing that EtOH induced changes only in AVP-expressing neurons located in the pPVN, and not mPVN [18]. Finally, the diuretic effects of EtOH are rapid and transient, making it unlikely to contribute to long lasting changes in AVP expression in the PVN.

Binge alcohol consumption among adolescents is a fundamental problem and mood disorders among both young and adult populations are increasing, therefore elucidating the long lasting consequences of binge EtOH exposure during adolescence is critical for understanding the aetiology of certain mood disorders. In this study we have identified long lasting effects of binge-pattern EtOH exposure during peri-puberty on the central regulators of the HPA axis, CRH and AVP, and on adult stress response. We have shown that adults who were exposed to a binge-pattern of alcohol during pubertal development have increased stress responses after subsequent alcohol exposure. Dysregulation of the HPA axis has been shown to be predictive of developing mood disorders however more experiments, such as introducing a novel stressor and measuring behavioural outcomes, are needed to determine if there is a direct link between binge-pattern alcohol exposure and the development of mood disorders. Overall, these data have identified yet another system and additional molecular targets that are affected long term by adolescent alcohol exposure and may lead to better understanding of factors that regulate HPA axis maturation during pubertal development.
